# Standardized multimodal intervention for stress-induced exhaustion disorder: an open trial in a clinical setting

**DOI:** 10.1186/s12888-020-02907-3

**Published:** 2020-11-05

**Authors:** Jakob Clason van de Leur, Monica Buhrman, Fredrik Åhs, Alexander Rozental, Gunilla Brodda Jansen

**Affiliations:** 1 Department of Psychology, Uppsala University, Von Kraemers allé 1A, SE-752 37 Uppsala, Sweden; 2 Department of Psychology and Social Work, Mid Sweden University, Kunskapens väg 1, SE- 831 40 Östersund, Sweden; 3 Department of Clinical Neuroscience, Karolinska Institute, Tomtebodavägen 18A, SE-171 76 Stockholm, Sweden; 4 Division of Rehabilitation Medicine, Department of Clinical Sciences, Karolinska Institute Danderyds University Hospital, SE-182 57 Stockholm, Sweden

**Keywords:** Stress-induced exhaustion disorder, Burnout, Long-term stress, Rehabilitation, Multimodal intervention, Negative effects

## Abstract

**Background:**

Long-term sick-leave due to stress-related ill-health is increasing in several economically developed countries. Even though different forms of interventions are administered in regular care for stress-related disorders, such as Stress-induced Exhaustion disorder (SED), the scientific evidence for the effectiveness of such treatments is sparse. The objective of this study was to explore changes in SED-symptoms and return-to-work-rates in a large group of SED-patients participating in a standardized Multimodal intervention (MMI) in a clinical setting.

**Method:**

This open clinical trial tracked 390 patients who fulfilled the criteria for SED undergoing a 24-week MMI, including return-to-work-strategies. Before inclusion, all patients underwent a multi-professional assessment by a team of licensed physicians, licensed psychologists, and licensed physiotherapists. Self-rated questionnaires were administered before treatment, at treatment-start, mid-treatment, post-treatment, and at 12-month follow-up. Within-group change was evaluated over time with mixed-effects models. Beyond different symptoms, working time, sick-leave compensation, and adverse effects were also measured.

**Results:**

There were significant improvements in symptoms of SED, burnout, anxiety, depression, and insomnia, with large within-group effect sizes (*d =* 0.91–1.76), improvements that were maintained at 12-month follow-up. Furthermore, there was a significant increase in quality of life and large improvements in average working time and sick-leave compensation. Some adverse effects were reported, mainly concerning an increase in stress, anxiety, and worry.

**Conclusion:**

SED-patients participating in this standardized MMI reported large symptom alleviation, increased working time and reduced sick-leave compensation, indicating a beneficial treatment. There were some adverse effects, but no more so than other psychological treatments. This study confirms previous findings that high levels of depression and anxiety decrease to sub-clinical levels during treatment, while symptoms of SED also decline, yet still persists above sub-clinical levels at 12-month follow-up. On the whole, this open clinical trial suggests that a standardized MMI, administered in a clinical setting, improves symptoms and return-to-work rates in a clinically representative SED-population.

**Trial registration:**

This study was registered on Clinicaltrials.gov 2017.12.02 (Identifier: NCT03360136).

## Background

During the last two decades, long-term sick-leave due to stress-related ill-health has been increasing in several economically developed countries [1–4]. Long-term exposure to non-traumatic stressors (such as deficiencies in the work environment, high work-load, divorce, socioeconomic difficulties, and interpersonal conflicts) without sufficient recovery can lead to a debilitating state of exhaustion characterized by physical fatigue, cognitive impairments and sleep disturbances [5, 6].

In the field of work-psychology, the term burnout is often used to describe the end-stage of a long-term stress process, where exhaustion is a cardinal symptom [7, 8]. The concept of burnout is, however, somewhat debated, and it has yet to be incorporated into any of the major diagnostic systems such as the Diagnostic and Statistical Manual of Mental Disorders (DSM-5) and the International Classification of Diseases, 10th edition (ICD-10 [9–12];). Because of this, as there is presently no internationally established terminology for stress-induced exhaustion, and different terms are used across different countries and disciplines, such as clinical burnout, job stress-related depression, exhaustion syndrome and neurasthenia [5, 13].

In Sweden, the diagnosis of “Stress-induced Exhaustion disorder” (SED) has been suggested to improve the identification and treatment of exhaustion due to long-term nontraumatic stress. SED (F43.8A) was accepted in the Swedish version of the ICD-10 in 2005 and can be regarded as an operationalization of severe “clinical burnout” [5]. Few studies have been published regarding the prevalence of SED, but in a recent cross-sectional study in northern Sweden, 4.2% of 3406 participants reported a physician-based diagnosis of SED [14]. Furthermore, there is a growing body of literature indicating that SED could be associated with structural brain changes and biochemical irregularities [15–17]. Even though the diagnosis of SED as of now is utilized primarily in Sweden, there have been several international publications that suggest that SED is not in any way a unique Swedish condition [4, 18–23].

The main symptoms of SED are severe physical and mental exhaustion, following a period of at least 6 months of stress-related exposure. This is accompanied by an increased need for recovery after mental efforts, cognitive impairments such as short-term memory loss, concentration difficulties, and sound- and light sensitivity. Other symptoms such as sleep disturbances, dizziness, nausea, headaches, gastrointestinal problems, and longstanding pain in the neck and shoulders are common [24, 25].

Multimodal interventions (MMI), where several interventions are administered simultaneously by a team of different professionals working together, was developed for the rehabilitation of longstanding pain [26]. More recently, MMI has been recommended for the treatment of SED. MMI should, according to clinical recommendations, include lifestyle changes concerning the balance between activation and recuperation, some relaxation techniques, psychotherapy (preferably in a group context), and specific return-to-work-interventions that include communication with the patient’s workplace. However, the empirical support for MMI in the treatment of SED is scarce [13].

One previous study has tracked the symptom development of SED-patients participating in MMR [24]. In this study, symptoms of depression and anxiety decreased rapidly within 3 months of treatment start, while symptoms of exhaustion declined more slowly, persisting to some extent 18 months later.

Two studies have investigated the effectiveness of MMI for SED [27, 28]. Both studies show within-group improvements over time in burnout symptoms and return-to-work in all groups, but few clinically significant differences are found between different treatments. It is also worth mentioning that existing studies of MMI for SED are seldom standardized, nor do they follow all of the clinical recommendations, making it harder to implement these treatments in clinical practice. In our understanding, previous studies of MMI for SED do not highlight or describe return-to-work-strategies as an integral core component of the rehabilitation process [24, 27, 28]. A few studies of unimodal interventions specifically targeting return-to-work in SED-patients have shown that the effects on return-to-work are very small or negligible, compared to regular Cognitive Behavioral Therapy (CBT), Acceptance and Commitment Therapy, or no treatment at all [29–32].

To sum up, even though MMI is recommended for SED, the current body of research speaks little as to whether it is effective or not, and how the contents of an MMI should be structured to facilitate symptom improvement and return-to-work.

Another potential shortcoming of previous research is the lack of questionnaires, specifically measuring the symptoms of SED. Since the diagnosis of SED is in its early stages, researchers have had to turn to measures of burnout. Most often, the Shirom-Melamed Burnout Questionaire (SMBQ) has been used. However, burnout and SED may not be interchangeable constructs. The SMBQ consists of four sub-scales (physical fatigue, cognitive weariness, tension, and listlessness) while SED emphasizes symptoms of exhaustion and includes some dimensions (recovery, memory, hypersensitivity to sensory impressions, the experience of demands) not necessarily captured by the SMBQ. As an example of this, Saboonchi, Perski, and Grossi [33] found that the variance in SED could not be explained by the concept of burnout assessed with the SMBQ in a population of SED. In 2014 the Karolinska Exhaustion Disorder Scale (KEDS) was developed explicitly measuring the construct of SED [34]. As of today, KEDS has not yet been evaluated in any clinical trial with a SED-population, even though the need for measuring the long-term symptoms of SED has frequently been emphasized [24, 35, 36].

When evaluating a treatment program such as MMI for SED, which is already being implemented in regular care despite the lack of adequate evidence, it is crucial to measure adverse effects. Even though many evidence-based psychological treatments show an overall good effect, only about half of the patients respond to treatment, and some even deteriorate [37, 38]. Despite a marked increase in research of psychological interventions during the past two decades, the adverse effects from these treatments have been widely neglected, and the need for more rigorous exploration is frequently advocated [39, 40].

Therefore, this open clinical trial had three aims: 1) To determine whether SED-patients participating in a standardized MMI report symptom alleviation through self-rating questionnaires measuring SED, burnout, insomnia, anxiety, depression, and quality of life; 2) to determine whether SED-patients participating in a standardized MMI report an increase in return-to-work-rates and 3) to evaluate the adverse effects of MMI for SED.

## Method

### Study design

This open clinical trial with a 12-month follow-up was part of a more extensive data collection from a standardized 24-week MMI for SED at two health care centers (PBM Sweden AB) in Stockholm, Sweden. These centers specialized in the rehabilitation of longstanding pain and SED and were part of a specialized health-care initiative called “The health care choice for treatment of longstanding pain with or without comorbidity, and Stress-induced Exhaustion disorder,” on behalf of Health Care Services Stockholm County. The clinics received referrals from general practitioners, primary health care centers, and occupational health services from all over Stockholm. This study was registered on Clinicaltrials.gov (Identifier: NCT03360136), approved by the Regional Ethical Review Board in Stockholm, Sweden (Approval Nr. 2016/1834–31/2) and followed the ethical principles of the Declaration of Helsinki.

### Participants and recruitment procedure

**Fig. 1:**
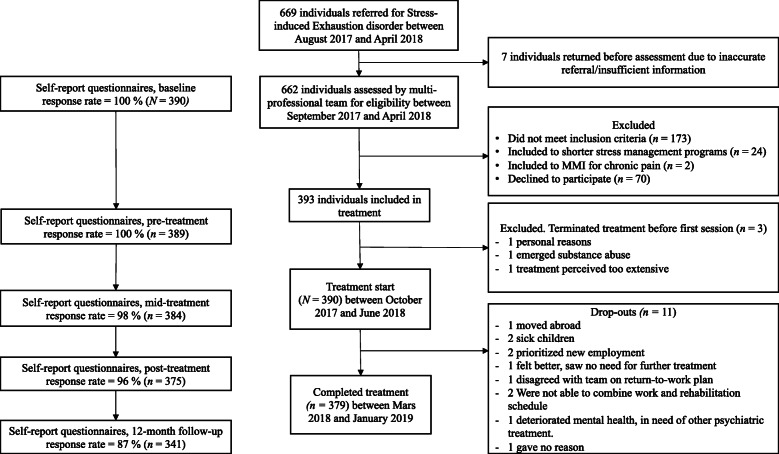
Flow of participants in the current study, together with reasons for dropping out throughout the trial

After being referred, the patients went through a multi-professional team assessment performed by a licensed physician (45 min), a licensed psychologist (60 min) and a licensed physiotherapist (45 min), together with a survey of baseline characteristics and several self-rating questionnaires (see measurements), after which a 30 min summarized assessment was returned to the patient by the team. A total number of 662 patients underwent a multi-professional assessment between September 2017 and April 2018. Out of these, 173 did not fulfill the inclusion criteria for participation in the MMI, two patients were included in the rehabilitation program for longstanding pain, and 24 were offered a short version of the rehabilitation program for SED (12 or 16 weeks) based on a clinical assessment that the 24-week MMI was too comprehensive in these cases (Fig. 1).

All SED-patients included in the 24-week MMI at the two units were asked to participate in the study. Of the 463 patients included in the 24-week treatment, 70 declined participation. Before treatment-start, three of the patients that had initially agreed to participate terminated their treatment and were excluded. A total of 390 patients were included in the study. Eleven of these dropped out during the treatment. Consequently, 379 patients completed the 24-week MMI.

### Inclusion and exclusion criteria

Inclusion criteria for the study were as follows: 1) referred for SED, fulfilled the criteria for SED; scored > 4.5 on the Shirom-Melamed Burnout Questionnaire (SMBQ; see section measurements for more information) together with a severity assessment: The patient described a substantial functional impairment due to symptoms of SED, severely limiting the ability to participate in both work and leisure time activities previously accustomed to before the onset of SED; 2)18–65 years of age; 3) the patient was considered to be suitable for group treatment and logistically able to participate in treatment; 4) no known abuse of alcohol or drugs; 5) did not participate in any other form of MMI. Psychiatric comorbidity was not an exclusion criterion per se, except severe depression, moderate/high risk of suicide, psychosis, or untreated PTSD. 6) Patients who reported a severe comorbid psychiatric or somatic illness deemed to be in more acute need of treatment than SED were also excluded. Since this was an open clinical study, no specific restrictions on medications were endorsed. For baseline characteristics, see Table 1.

**Table 1:** Baseline characteristics of patients with Stress-induced Exhaustion disorder (*N* = 390) participating in a 24-week Multimodal intervention

Characteristics	Mean	SD
Age	43.69	9.42
	*n*	%
Sex		
- Female	344	88
- Male	46	12
Marital status		
- Single or other	131	33
- Married/living together	238	61
- Partner (living apart)	21	5
Education		
- Elementary school and/or secondary school	92	23
- University < 3 years	62	16
- University ≥3 years	191	49
- Other	45	12
Nationality		
- Sweden	337	86
- European	21	6
- Other	32	8
Approved sick-leave compensation		
- 0%	99	25
- 25%	13	3
- 50%	60	15
- 75%	36	9
- 100%	182	47
Working time (including studies)		
- 0%	232	59
- 1–25%	34	9
- 26–50%	71	18
- 51–75%	16	4
- 76–100%	37	9
Occupational status		
- Employed/self-employed	345	89
- Studying	20	5
- Unemployed	25	6
Symptom-duration before seeking treatment		
- 1–12 months	149	38
- > 12 months	241	62
Previosly on sick-leave due to Stress-induced Exhaustion disorder	137	35
Comorbidity		
- Suffers from some kind of physical pain	257	66
- Describes longstanding pain	48	12
- Number of patients with only Stress-induced Exhaustion disorder	217	56
- Psychiatric comorbidity	155	40
- Somatic comorbidity	27	7
- Number of patients with more than one comorbid diagnosis	32	8
- Number of patients with both somatic and psychiatric comorbidity	15	4
Medications		
- Psycholeptic sleep medication	103	26
- Psychoanaleptic antidepressant medication	158	41
- Psycholeptic sedative medication	72	18
- Other, incl. Pain medication, paracetamol/NSAID and medications not prescribed by a physician	254	65

### Treatment

**Fig. 2:**
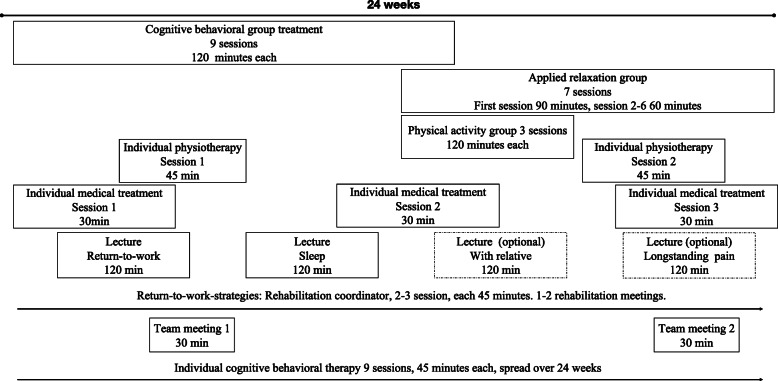
Overview of the 24-week Multimodal intervention for Stress-induced Exhaustion disorder in the current study

Legend: Overview of the 24-week Multimodal intervention for Stress-induced Exhaustion.

After inclusion, the patients received a schedule of all appointments of the standardized team-based MMI, spanning over 24 weeks. Each multi-professional team consisted of one licensed psychologist, one licensed M.D., one licensed physiotherapist, and one rehabilitation coordinator (occupational therapist, licensed psychologist, or licensed nurse) responsible for overseeing return-to-work-strategies. Although the content of the rehabilitation program and the order of the group treatments were standardized, the timing of the individual sessions, team meetings, and rehabilitation meetings could vary somewhat. For an overview of the treatment components, see Fig. 2. A more extensive outline of each component, together with the contents of each group treatment session, is supplied in the online supplement.

### Measurements

Baseline characteristics and measures of psychological variables were collected during the assessment phase, at the start of rehabilitation, mid-treatment, post-treatment, and at 12-month follow-up. All questionnaires were administered digitally through a secure online-login, which is a reliable way of collecting psychological self-report measures [41]. To decrease the risk of instrumentation-bias, the order of all self-rating questionnaires was randomized at each instance of administration.

#### Primary outcomes

SED symptoms were measured by the Karolinska Exhaustion Disorder Scale (KEDS), which is explicitly constructed for measuring symptoms of SED [34]. KEDS consists of nine items rated on a 7-point Likert scale ranging from zero to six. The items correspond to the diagnostic criteria of SED and are formulated in line with autobiographical descriptions from patients suffering from SED. At scores 0, 2, 4, and 6 on each item, different definitions are presented underneath. A score of 19 or above indicates “at risk of SED.” KEDS has demonstrated good internal consistency with a reported Cronbach’s alpha of .94 and has been shown to discriminate between SED-patients and healthy controls effectively. Furthermore, it is sensitive to measure changes during treatment (ibid.). Cronbach’s alpha for KEDS in this study was .75.

Working time was evaluated through the self-rated question: “How much are you working right now?” answered in percent. Only participants with some form of occupation (employed or studying; *n* = 365) were included in the return-to-work calculations. Additionally, the self-rated question “How much compensation are you receiving from the Swedish Insurance Agency?” was administered to evaluate sick-leave compensation, answered in percent. Data from the Swedish Social Insurance Agency is to be gathered in the future and will be published elsewhere.

#### Secondary outcomes

Shirom-Melamed Burnout Questionnaire (SMBQ) was used to measure burnout [42]. SMBQ consists of 22 items rated on a 7-point Likert scale varying from one (almost never) to seven (almost always). The scale consists of four subscales (physical fatigue, cognitive weariness, tension, and listlessness), and an overall index is calculated from the mean of all items. The SMBQ was initially developed to be used in working populations where a score of ≥3.75 has been suggested as indicative of a high degree of burnout [43]. Later the SMBQ has been validated as a measure of burnout in clinical populations, suggesting a 4.4 cut-off for SED [44]. To ensure the severity of patients included within this health care imitative mentioned above (see Study design), the Health Care Services Stockholm County increased this score to 4.5. Therefore 4.5 was used as a cut-off in the current study. Cronbach’s alpha for SMBQ in this study was .87.

The Hospital Anxiety and Depression Scale (HADS) was used to measure symptoms of anxiety and symptoms of depression [45]. HADS comprises of two sets of 7-item sub-scales rated on a 4-point Likert scale ranging from zero to three (with different statements for each item), measuring anxiety and depression. Each subscale has a total score of 21, where a score of ≥11 indicates a probable caseness of a mood or anxiety disorder. In a review of 47 articles, HADS has been shown to have satisfactory internal consistency with a mean Cronbach’s alpha of 0.83 for HADS-A and 0.82 for HADS-D, and to be a valid measure of change over time in depression and anxiety in relation to numerous treatments [46]. The Cronbach’s alpha in this study was .79 for the anxiety scale and .78 for the depression scale.

Insomnia Severity Index (ISI) was used to assess problems with insomnia since sleep has been shown to be a predictor of return-to-work in patients receiving treatment for SED [47, 48]. ISI is a widely used measure in the research of psychological treatment for sleeping disorders. It has demonstrated adequate internal consistency and proven to be a reliable measure of changes in sleep during treatment [47]. Cronbach’s alpha for ISI in this study was .84.

EuroQol five dimension scale (EQ-5D) was used to measure health-related quality of life [49]. The scale consists of five questions (mobility, self-care, usual activities, pain/discomfort, and anxiety/depression), each rated on a severity level of three (1 = no problems, 2 = some problems, 3 = severe problems). The answers are measured against an index tariff based on a sample from the population. Here, the standard procedure of using the UK EQ-5D index tariff was facilitated to obtain values for the health states [50].

#### Negative effects

The Negative Effects Questionnaire (NEQ) was used to measure adverse events of treatment. It consists of 32 items rated on a 5-point Likert scale ranging from “not at all” to “extremely so,” with a factor structure of six: symptoms, quality, dependency, stigma, hopelessness, and failure [39]. In conjunction with each item, the patient also decides whether or not he/she has experienced the adverse event at all, and whether it could be attributed to the treatment or other external circumstances. The scale has been shown to have good reliability and to effectively measure the adverse effects of psychological interventions [51]. While the NEQ is developed to measure adverse events in psychological treatments, the current treatment was multimodal. Since the MMI mainly consisted of psychological interventions, the NEQ was still deemed as a valid measure of adverse events.

#### Treatment credibility

The Treatment Credibility Questionnaire (TCQ) was administered at the start of treatment and mid-treatment (10 weeks after treatment start) to assess the credibility of the treatment. The TCQ has demonstrated high internal consistency and good test-retest reliability [52].

### Statistics

Statistical analysis was performed in IBM SPSS Statistic version 25 (IBM Inc., Armonk, NY, USA). All repeated symptom outcome measures were analyzed using mixed-effects models, with time as a fixed effect and random intercepts. Model fit was evaluated using – 2 restricted log-likelihood. Data were analyzed using an intention-to-treat procedure, meaning all patients that started treatment were included in the analysis, irrespective of completion. Missing data were handled using maximum likelihood estimation. Pearson’s χ^2^-tests and independent samples t-tests were used to analyze potential differences between completers and drop-outs.

We calculated within-group effect sizes over time using Cohen’s *d*, where 0.2–0.5 was considered small, 0.5–0.8 moderate, and > 0.8 large [53]. The guidelines by Jacobson and Truax [54] were utilized to evaluate clinically significant change. To meet the criteria for clinically significant change on KEDS, the participants had to demonstrate a reliable change of 8.72 and meet the criteria for clinically significant improvement. The cut off of 19 on KEDS was chosen, as this indicates “at risk of SED,” based on a sample of 117 healthy individuals [34]. There are, however, currently no established criteria for clinically significant improvement in the treatment of SED, and the score of 19 has been questioned and deemed as too low for diagnostic purposes in clinical settings [20]. Because of this, complimentary analysis of clinically significant change was also performed on SMBQ to give a more balanced view of the observed improvements. On SMBQ, participants had to demonstrate a reliable change of 0.69 and score under the cut off of 4.4, based on the recommendations of Lundgren-Nilsson, Jonsdottir, Pallant & Ahlborg [44]. Patients who did not complete post-treatment measures, as well as 12 months follow-up, were categorized as not clinically significantly improved. Furthermore, patients who reported an increase of 8.72 on KEDS at the end of treatment were categorized as deteriorated.

## Results

### Adherence and attrition

Of the 390 patients included, 3% (*n* = 11) dropped out during the rehabilitation period (Fig. 1). There was a significantly lower level of education in the drop-out group (χ^2^ (4) = 9.59, *p* < .05) compared to completers. There was also a significantly higher degree of insomnia measured with ISI in the population that dropped out (*M* = 20.09, *SD* = 5.58) compared to the patients who completed the treatment (*M* = 16.27, *SD* = 5.85); *t* (388) = 2.14, *p* < .05. Full characteristics for drop-outs compared with completers are presented in the online supplement.

On average the length of MMR was 23.99 weeks (SD ± 2.85). Average number of completed sessions were as following: Cognitive-behavioral group treatment 7.91 (SD ± 1.23) of 9; Applied relaxation group 5.36 (SD ± 1.81) of 7; Physical activity group 1.97 (SD ± 0.99) of 3; Individual CBT 9.73 (SD ± 0.72) of 10 (including 1 booster session); Individual M.D. 3.41 (SD ± 0.72) of 3; Individual Physiotherapist 1.95 (SD ± 0.76) of 2; Individual rehabilitation coordinator 1.48 (SD ± 0.95) of 2–3, Team meetings 1.97 (SD ± 0.36) of 2; Rehabilitation meetings 1.44 (SD ± 1.03) of 1–2; Sleep lecture 0.92 (SD ± 0.28) of 1; Lecture on return-to-work-process 0.83 (SD ± 0.38) of 1; Longstanding pain lecture 0.37 (SD ± 0.48) of 1 (optional); Lecture with relative 0.33 (SD ± 0.48) of 1 (optional).

### Primary outcome measures

**Fig. 3:**
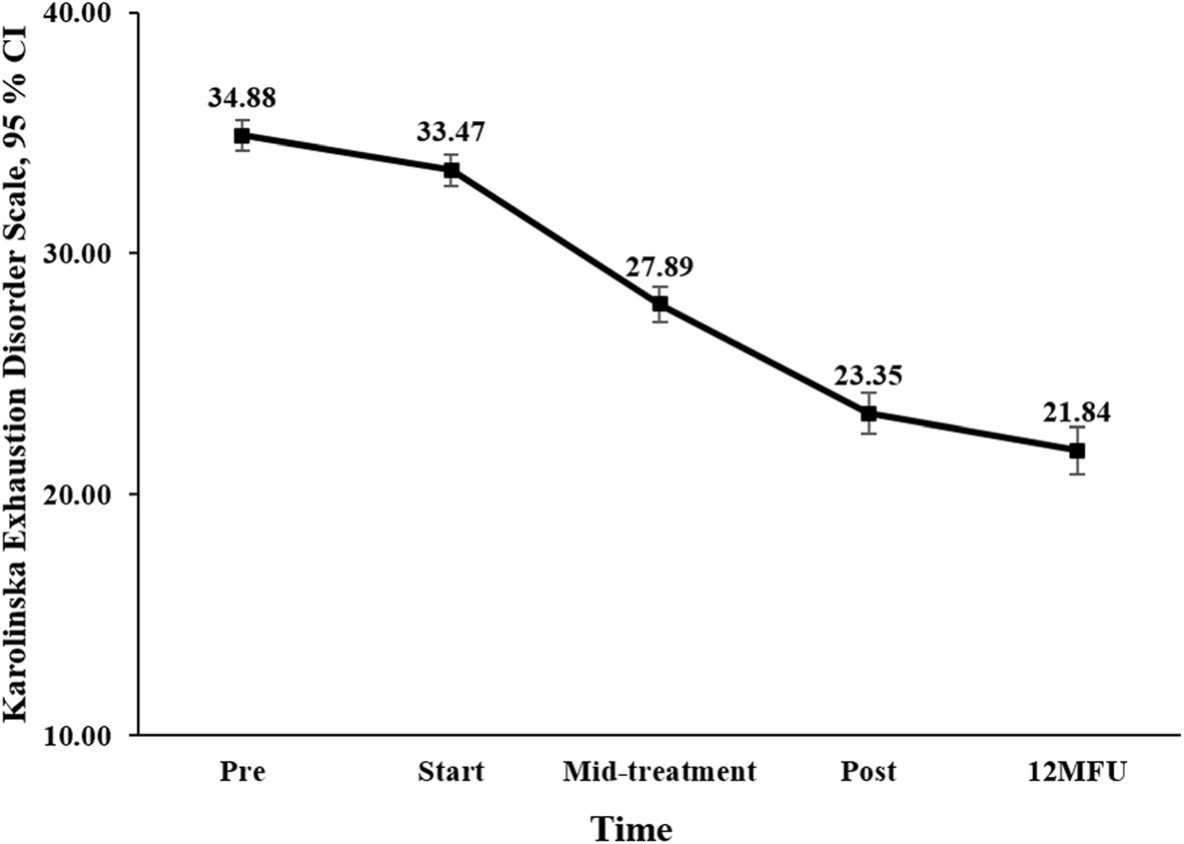
Changes in mean scores (intention-to-treat procedure) on Karolinska Exhaustion Disorder Scale in patients with Stress-induced Exhaustion disorder (*N* = 390) participating in a 24-week Multimodal intervention. Pre, before treatment; Start, treatment start; Mid-treatment, 12 weeks into treatment; Post, after treatment; 12MFU, 12-month follow-up

There was a significant improvement over time on KEDS, *F* (2, 360.94) = 445.04, *p* < .01 with a large within-group effect size at post-treatment (*d* = 1.61), which was maintained at 12-month follow-up. Means, standard deviations, and effect sizes are presented in Table 2. Figure 3 shows the change in KEDS over the course of treatment.

**Table 2:** Means, return-to-work-rates and within-group effects sizes (Cohen’s *d*) of symptoms over time in patients with Stress-induced Exhaustion disorder (*N* = 390) participating in a 24-week Multimodal intervention

Measure	Mean (SD)			Within-group effect size	
	Pre	Post	12MFU	pre-post	pre-12MFU
KEDS	34.88 (6.18)	23.35 (8.09)	21.84 (9.13)	1.61	1.69
Working time* %	26 (34.4)	52 (29.92)	76 (34.31)		
SLC %	62 (41.7)	39 (31.7)	12 (29.9)		
HADS					
- anxiety	11.25 (4.07)	6.92 (3.64)	6.71 (3.86)	1.12	1.15
- depression	11.22 (3.84)	6.50 (3.92)	6.26 (4.30)	1.22	1.23
SMBQ	5.63 (0.63)	4.00 (1.11)	3.87 (1.24)	1.75	1.76
ISI	16.38 (5.87)	9.92 (5.75)	9.81 (6.06)	1.11	1.10
EQ 5D	0.61 (0.16)	0.75 (0.14)	0.76 (0.17)	0.91	0.92

*Pre* before treatment; *Post* after treatment; *12MFU* 12-month follow-up; *KEDS* Karolinska Exhaustion Disorder Scale; *SLC* Sick-leave compensation; *SMBQ* Shirom-Melamed Burnout Questionnaire; *HADS* Hospital Anxiety and Depression Scale; *ISI* Insomnia Severity Index; *EQ-5D* EuroQol five dimension scale. *Patients with some form of occupation, employed or studying (*n* = 365)

At post-treatment, there was an increase in average working time with 26% (where 100% is full time-employment) in patients with some form of occupation (employed or studying; *n* = 365), as well as a decrease in sick-leave compensation with 23% (where 100% is full-time sick-leave compensation) in the sample as a whole (*N* = 390). At 12-month follow-up there was a 50% increase in average working time in patients with some form of occupation (employed or studying *n* = 365) and a 49% decrease in sick-leave compensation in the sample as a whole (*N* = 390). Means and standard deviations are presented in Table 3. To illustrate: If a participant worked 25% at the start of treatment and received 75% sick-leave compensation, at 12-month follow-up, this participant could be expected to work 75% (25% + 50%) and received a sick-leave compensation of 26% (75 - 49%). A more extensive overview of the changes in the distribution of working time and sick-leave compensation is presented in the online supplement.

### Secondary outcome measures

**Fig. 4:**
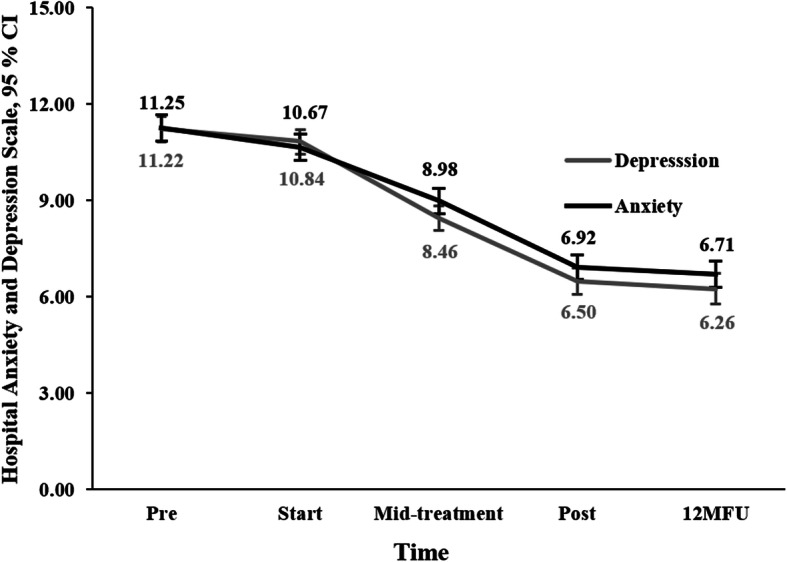
Changes in mean scores (intention-to-treat procedure) on the subscales depression and anxiety of the Hospital Anxiety and Depression Scale, in patients with Stress-induced Exhaustion disorder (*N* = 390) participating in a 24-week Multimodal intervention. Pre, before treatment; Start, treatment start; Mid-treatment, 12 weeks into treatment; Post, after treatment; 12MFU, 12-month follow-up

There was a significant improvement over time on HADS depression *F* (2, 356.00) = 297.98, *p* < .01, HADS anxiety *F* (2, 366.50) = 255.04, *p* < .01, SMBQ, *F* (2, 354.81) = 551.18, *p* < .01, ISI *F* (2, 365.22) = 254.44, *p* < .01 and EQ. 5D *F* (2, 366.67) = 151.80, *p* < .01, with overall large within-group effects sizes at post-treatment (*d =* 0.91–1.75). All these improvements were maintained at 12-month follow-up. Means, standard deviation, and effect sizes are presented in Table 2. Figure 4 shows changes in mean scores over time on HADS for both subscales of depression and anxiety.

### Clinical significant change

At the end of treatment, the proportion of patients fulfilling the criteria for clinically significant change on KEDS was 27% (*n* = 106). At 12-month follow-up, this change was increased to 37% (*n* = 143). On SMBQ, the proportion of patients fulfilling the criteria for clinically significant change was 57% (*n* = 222), which was maintained at 57% (*n* = 223) at 12-month follow-up.

### Negative effects

In total, 57% (*n* = 224) reported some form of adverse effect attributed to treatment, mainly concerning an increase in stress, anxiety, and worry. The most frequently reported question was, “I felt like I was under more stress” at 26% (*n* = 97). No participant reported an increase of 8.72 of their KEDS score post-treatment compared to their baseline score, showing a deterioration rate of 0% (*n* = 0).

## Discussion

This open clinical trial explored changes in symptoms and return-to-work-rates in 390 SED-patients participating in a standardized MMI in a clinical setting. It also evaluated the negative effects of said treatment. In general, there were significant and large improvements in all measures. Patients showed reduced symptoms of SED, burnout, insomnia as well as anxiety and depression following treatment, with large within-group effect sizes at 12-month follow-up (*d =* 0.91–1.76). Patients also reported a significant increase in quality of life. At 12-month follow-up, 37% (*n* = 143) of the patients achieved clinically significant change on KEDS and 57% (*n* = 223) measured by SMBQ. Beyond these improvements, patients with some form of occupation (employed or studying; *n* = 365) had returned to work with an average of 50%, and in the sample as a whole (*N* = 390) sick-leave compensation was reduced by 49% at 12-month follow-up. While a few patients reported adverse effects of treatment, mainly concerning an increase in symptoms of stress, the overall impression is that SED-patients participating in a standardized MMI reported symptom alleviation, an increase in working time, a decrease in sick-leave compensation, and enhanced quality of life.

### Changes in symptoms

The reduction in burnout was comparable to or larger than with previous studies [24, 27, 28]. While no causal inferences can be made, these improvements are promising given that the MMI extends over 12 months in Stenlund et al. [28], and 18 months in Glise et al. [24], compared to 6 months in the current study.

Continuous measurements of burnout, anxiety, and depression have been administered to a large SED-population once before. In Glise et al. [24], a marked decrease in anxiety and depression (measured with HADS) was observed after 3 months of treatment, after which levels continued to decrease further to subclinical levels. Meanwhile, symptoms of burnout steadily declined, yet remained above sub-clinical levels at an 18-month follow-up. In the current study, the same pattern was recognized. Here, SED-patients reported a high degree of comorbid symptoms of anxiety and depression at the start of treatment. These symptoms successively decreased as treatment was initiated, while symptoms of burnout (and in this case also SED) declined, but still persisted to a higher degree at 12-month follow-up (21.8 on KEDS and 3.87 on SMBQ). Because a score of ≥19 on KEDS is indicative of SED, and a score of ≥3.75 is indicative of a “high degree of burnout” [34, 43], the symptoms of SED that remain are, despite being substantially improved, still above sub-clinical levels.

Patients in the current study reported a decrease in insomnia with a large effects-size, which was maintained at the 12-month follow-up. This decrease is encouraging, given that improved sleep quality has been shown to play a vital role in the effective treatment of SED [6, 48, 55].

### Return to work

Concerning return-to-work rates, one previous study of MMI for SED has reported return-to-work-numbers before and after treatment. In the study by Stenlund, Nordin, and Järvholm [56], participants reported an average of 72% on full-time sick-leave at baseline, 60% at the end of treatment (12 months later), and 38% at 12-month follow-up (personal communication with the author).

The sick-leave rates in the current study are markedly lower than in Stenlund, Nordin, and Järvholm [56]. These differences could, of course, mirror differences in the administration of sick-leave insurance, which is affected by factors such as changes in political policies over time and varying regional bureaucratic traditions. They could also be a reflection of dissimilarities in the number of sick days before starting treatment in the above-mentioned populations. Because of this, comparisons with the current study, of course, can not tell us anything about which of these interventions is more effective. What it can tell us is that the positive return-to-work-rates in the current study may be interpreted as an indication of an effective return-to-work-intervention.

### Negative effects

To our knowledge, this is the first trial where the NEQ has been used in face-to-face clinical treatment, and the adverse effects do not appear to stand out. Compared to large samples of patients receiving CBT administered via the internet and a survey among respondents from a psychiatric population receiving psychological treatment, the adverse effects are comparable to or lower than in the current study [57]. Also, rates of deterioration are lower compared to the general psychiatric and primary care populations in Sweden [58, 59]. These finding, together with the relatively low rate of drop-outs (3%), implies that this MMI does not seem to pose a higher risk of harm than other unimodal psychological treatment strategies.

### Limitations and strengths

There are several strengths and weaknesses in the current study that need to be highlighted. First and foremost, this open clinical trial is not controlled, which impedes any causal inferences between observed changes and the treatment administered. However, the duration of symptoms of SED before starting treatment varied across participants, which - together with the comparisons of SMBQ in studies made previously - indicate that the observed changes are probably not exclusively the result of the natural course of SED, spontaneous improvement, or regression to the mean.

Even though the treatment was standardized, there are still some parts of the treatment that were not (the content of the individual sessions). This limitation reduces the internal and external validity of this study. Another limitation is the exclusive use of self-report measures. An improvement would have been to use another form of outcome procedures, such as standardized clinician ratings. It would, of course, also have been more reliable to evaluate return-to-work through other measures than self-rating-questionnaires. One option is to retrieve actual sick-leave data from the Swedish Social Insurance Agency, which will be carried out in a later publication.

As far as we know, this population is the largest population of SED-patients to date, to have been tracked through an intervention, ensuring a high power and clinically representative population. All patients were medically examined before treatment and were diagnosed by a team of professionals, including a licensed physician and a licensed psychologist. Thanks to this procedure, comorbid diagnosis, and medications, including Anatomical Therapeutic Chemical Classification (ATC-codes), are accounted for. Furthermore, the order of all questionnaires was randomized at each instance of administration to reduce the risk of instrumentation bias. Another strength is the use of a standardized treatment with thoroughly described treatment content. Even though we cannot infer any change from treatment, we can at least conclude that overall, all patients received the same treatment, except for some content in the individual sessions.

The fact that the study was set at two different locations in Stockholm and had a large number of clinicians delivering the treatment increases the generalizability of the study and significantly increases the natural and external validity, as well as decreases the risk of therapist-bias.

Despite being set in a naturalistic clinical context, the drop-out rate is low, and the response rate is relatively high for this setting. This further increases generalizability of the data to other settings. It can be assumed that the patients, overall, perceived the intervention as relevant. Lastly, there is a low exclusion rate in the recruitment phase, ensuring that the sample treated in the current study can be considered clinically representative of an actual SED-population.

### Future research

As far as we know, most existing published trials compare variations of the same MMI, and no study has yet compared MMI to a wait-list-control. Therefore, there is an apparent need for randomized controlled trials to ascertain the efficacy of MMI for SED. Beyond this, there is a need to differentiate what components of MMI lead to symptom-improvement. MMI is the recommended modality of treatment for SED, even though the evidence is scarce. It would be interesting to compare a full MMI to a less cumbersome, unimodal treatment, such as an individual CBT or individual physiotherapy with physical exercise, or just return-to-work-strategies. Since the most substantial symptom improvement shown to date in SED-patients resulted from an internet-delivered treatment [60], looking at other modes of treatment besides MMI should be encouraged. This is especially true considering the administration of MMI can be logistically cumbersome and resource-demanding, with the risk of decreasing access to treatment.

Other treatment protocols of common mental disorders (CMD), such as behavioral activation for depression, or Clark’s treatment for social anxiety, rest upon a theoretically founded framework of how the condition arises and is maintained [61, 62]. An equivalent theoretical framework for the cause and maintenance of SED is currently missing in the clinical treatment literature. Therefore, it is relevant to explore potential predictors and process variables to increase understanding of the theoretical conceptualization and treatment of SED.

A few observations about the sample are also worth mentioning: 40% (*n* = 155) are diagnosed with other psychiatric diagnosis, 41% (*n* = 158) are on antidepressant medications, and 66% (*n* = 257) describe some form of comorbid pain. Furthermore, 35% (*n* = 137) of the patients reported previously being on sick leave due to SED. In line with previous studies [24, 25], these observations highlight a considerable burden of disease regarding both mental and somatic symptoms in SED-patients, which need to be accounted for in clinical care and further explored in future research.

There was a considerable discrepancy in the number of participants achieving clinically significant change when measured by KEDS compared to SMBQ. This indicates that SMBQ and KEDS are not mutually overlapping questionnaires, and probably do measure different underlying constructs, in accordance with previous research [33]. Because of this, the differences between SMBQ and KEDS should to be explored more thoroughly in future research.

One last thing to note is that regardless of how clinically significant change was calculated, a large proportion of the population did not reach a clinically significant change at 12-months follow up (63% based on KEDS, 43% based on SMBQ). This, together with the relative increase in variation in the KEDS measurements at post- and 12-month follow-up, compared to the pre-measurements, suggest that different patients fare differently in treatment. Little is still known about the population with SED. Therefore, future research should explore the possibilities of sub-groups through cluster analysis.

## Conclusions

SED-patients participating in this standardized MMI reported large symptom alleviation, increased working time, and reduced sick-leave compensation, suggesting that they did seem to benefit from treatment. While no causal inferences can be made, comparisons with previously published treatment studies indicate that observed improvements, in part, are attributable to treatment. Furthermore, this study confirms previous findings that high levels of depression and anxiety decrease to sub-clinical levels during treatment, while symptoms of SED also decline, yet still persists above sub-clinical levels at 12-month follow-up. There were some negative effects, but no more so than other psychological treatments. In conclusion, findings suggest that a standardized MMI, administered in a clinical setting, improves symptoms and return-to-work-rates in a clinically representative SED-population.

## Supplementary information

**Additional file 1.** Treatment overview supplement.

**Additional file 2.** Extended demography supplement.

**Additional file 3.** Supplementary figures of return-to-work-rates.

## Data Availability

The datasets generated and analyzed during the current study are not publicly available because this data is part of a larger data-collection that is currently ongoing. The data is available from the corresponding author on a reasonable request.

## Abbreviations

CBT: Cognitive Behavioral Therapy
ISI: Insomnia Severity Index
KEDS: Karolinska Exhaustion Disorder Scale
MMI: Multimodal Intervention
SMBQ: Shirom-Melamed Burnout Questionnaire
SED: Stress-induced Exhaustion disorder
HADS: The Hospital Anxiety and Depression Scale
TCQ: The Treatment Credibility Questionnaire
