# Supplementary material for: Standardized multimodal intervention for stress-induced exhaustion disorder: an open trial in a clinical setting
Source: BMC Psychiatry. 2020 Nov 5;20:526. doi: 10.1186/s12888-020-02907-3 (PMC7643309; doi:10.1186/s12888-020-02907-3)
Supplement: Supplementary file 1 — Additional file 1. Treatment overview supplement. [file 12888_2020_2907_MOESM1_ESM.docx]

TREATMENT OVERVIEW SUPPLEMENT

The MMI was comprised of the following components:

- *Cognitive-behavioral group treatment:* A cognitive-behavioral group treatment focusing on behavioral changes to reduce the risk of long-term stress-related symptoms. The treatment comprised of nine weekly sessions (120 minutes) with eight to nine participants.
- *Applied relaxation group*: This group treatment was based on a shortened version of Applied relaxation [1], coupled with training in deep breathing, comprising of 7 sessions (first session 90 minutes, the remaining 60). The contents of each session are supplied in the online supplement.
- *Physical activity group*: Three weekly sessions (120 minutes) promoting adjusted increased physical activation, starting at the end of the cognitive-behavioral group treatment. The contents of each session are supplied in the online supplement.
- *Individual physiotherapy*: Two sessions (45 minutes) with a licensed physiotherapist to promote physical activation and relaxation, following the Swedish guidelines "Physical Activity in the Prevention and Treatment of Disease" (FYSS).
- *Individual medical treatment*: Three sessions (30 minutes) to review and discuss sick-leave, medication, and diagnostic evaluation.
- *Lectures:* Four lectures (120 minutes) were offered to all participants. The first two were mandatory, consisting of one lecture about sleep (psychoeducation about sleep, sleep medications, behavioral sleep hygiene recommendations based on the principles of stimulus control), and one on the return-to-work-process (regulatory framework of the sick-leave insurance system, information about return-to-work-strategies in the MMI, etc.). Also, the patients were offered two voluntary lectures: One attended with relatives, focusing on essential information about SED and the content of the MMI; One about the interaction between long-term stress and pain, and a brief review of basic coping strategies for longstanding pain.
- *Return-to-work*: Two to three sessions (45 minutes) with a rehabilitation coordinator focusing on return-to-work-strategies. Rehabilitation meetings with the rehabilitation coordinator (and, if needed, the patient's psychologist) were conducted with the patient's employer at one or two (or in rare cases, three) occasions. The purpose of these meetings was to discuss work-adjustments concerning SED and to establish a viable plan for return-to-work. If the patient was not employed, other vocational measures were taken, for example, meetings the Swedish Social Insurance Agency and the Swedish Public Employment Service.
- *Team meetings:* Two sessions (30 minutes) together with at least two of the four-team members. The first meeting took place six to eight weeks after the rehabilitation started and the second at the end of treatment. The purpose of these meetings was to reconcile the rehabilitation process; set treatment goals, evaluate treatment progress, plan and discuss the return-to-work process, and discuss further recommendations.
- *Individual CBT*: Nine individual sessions (45 minutes) of CBT focusing on supporting participation in the group treatments as well as helping the patients with their personal behavioral change needs. The content of these sessions was not standardized but instead based on an individual assessment made by the psychologist. If the patient had comorbid psychiatric problems such as depression, insomnia, panic disorder, or generalized anxiety disorder, limited interventions focusing on these problems (for example, interoceptive exposure, behavioral activation, worry journal) was implemented to help the patient assimilate the rest of the MMI-content. The participants did not necessarily have the same psychologist in the individual CBT as in the cognitive-behavioral group treatment.

| **Cognitive-behavioral group treatment** | | | | |
| --- | --- | --- | --- | --- |
| Session | Profession | Content | Purpose | Home Assignment |
| 1 | PS, MD | *Introduction*  Psychoeducation about stress, exhaustion disorder, and recuperation behaviors. | Normalize temporary stress-reactions as an inevitable and vital part of human life, see the difference between acute stress and symptoms of long-term stress. Instill hope and convey the importance of prioritizing recuperating behaviors, rather than eliminating all stressors in life. | Chart the balance between stressors and recuperation. Identify and reflect upon current, previous and potential recuperation behaviors. Do one recuperating behavior. |
|  |  |  |  |  |
| 2 | PS | *Stress behaviors* How behavioral change is carried out with the help of functional analysis. Behavioral change of common stress behaviors. | Learn basic principles of behavioral change and apply them to one's stress behaviors. | Practice behavioral change through the reversal of at least one stress behavior, by facilitating so-called "slow behavior" (in example walking slow or doing one thing at a time). |
|  |  |  |  |  |
| 3 | PS, PH | *Valued direction* | Help the patient identify life-values, promote acceptance of unpleasant experiences and reinforce behavioral change in a valued direction. | Identify values, take one step (behavioral change) in a valued direction. |
|  |  |  |  |  |
| 4 | PS | *Emotions*  The evolutionary function of different emotions and their influence on behavior. A rationale for mindfulness. | How to cope with emotions as obstacles standing in the way of change one's valued action. Practice mindfulness. Increase acceptance of unpleasant experiences, decrease emotional avoidance, and discriminate when emotional- and stress-related avoidance is helpful and not. | Practice mindfulness and identify experiences and impulses of behaviors in one context that triggers strong emotions. |
|  |  |  |  |  |
| 5 | PS | *Thoughts*  Rationale for thoughts and how they influence behavior. Rationale for exposure interventions. | Learn how to cope with catastrophizing and negative thoughts as obstacles standing in the way of change in one's valued action. Promote acceptance of the existence of negative thoughts, that thoughts are just thoughts and are hard to explain. Learn the principles of exposure treatment. | Practice a non-judgmental approach to negative thoughts via mindfulness. Plan and perform one exposure exercise in relation to a valued direction. |
|  |  |  |  |  |
| 6 | PS, PH | *Physical exercise* Psychoeducation about the overall positive effects of exercise and the specific impact on stress-related ill-health. How to engage in physical activity when suffering from exhaustion disorder. | Promote the importance of physical exercise and daily physical activation. A significant focus was also to communicate that exercise is not dangerous when exhausted, and how the patients could adjust their training in compliance with current bodily prerequisites. | Try a workout in a way that challenges one's previous exercise behaviors (for example, if one is careful, push harder. Or if one always pushes oneself to the limit, try to do a workout at 60 % of maximum intensity). |
|  |  |  |  |  |
| 7 | PS | *Perfectionistic behaviors* Definition of clinical perfectionism and how to change perfectionistic behaviors through exposure exercises. | To reflect upon personal perfectionistic behaviors and high standards, and to formulate ideas for idiosyncratic exposure interventions. | Implement one exposure exercise in relation to one's perfectionistic behavior. |
|  |  |  |  |  |
| 8 | PS | *Assertiveness and communication* | Practice asking for help, set boundaries, and exert assertive statements of one's needs. | Translate assertiveness skill into one situation previously avoided, through an exposure exercise. |
|  |  |  |  |  |
| 9 | PS | *Summary of treatment and maintenance plan* | Repeat treatment content and establish a maintenance plan to decrease the risk of relapse in exhaustion-related behaviors. | Maintenance plan |
| MD, Medical Doctor; PS, Psychologist; PH, Physiotherapist | | | | |
|  |  |  |  |  |
|  | | | | |
| **Applied relaxation group** | | | | |
| Session | Profession | Content | Purpose | Home Assignment |
| 1 | PS | Introduction to applied relaxation, rationale for treatment and long progressive relaxation (LPR) part one | Understand the relation between relaxation and acute stress and practice LPR part one. | LPR part 1 two times / day |
|  |  |  |  |  |
| 2 | PS | LPR part 2 | Practice LPR part 2, manage challenges and difficulties with relaxation. | LPR part 2 two times / day |
|  |  |  |  |  |
| 3 | PS | Stomach breathing, Short progressive relaxation (SPE) and psychoeducation concerning the interaction between the respiratory system and the stress reaction. | | SPE two times / day. Stomach breathing 10 minutes / day |
|  |  |  |  |  |
| 4 | PS | Slow stomach breathing (5-6 breaths/minute) and Conditioned relaxation (CR) | Practice slow stomach breathing, decrease time to achieve relaxation. | CR two times / day. Slow stomach breathing 10 minutes / day |
|  |  |  |  |  |
| 5 | PS, PH | Differential relaxation (DR) | Increase awareness of the tension in different muscles during activity, practice differential relaxation to maintain relaxation during movement. Decrease time to relax. | DR two times/day, only use essential muscles during one walk every day and during one activity (for example drinking coffee, speaking on the phone) |
|  |  |  |  |  |
| 6 | PS | Fast relaxation | Practice fast relaxation and map everyday situations (both neutral and stressful) when SA should be applied | SA 20 times per day in neutral situations and when stressed |
|  |  |  |  |  |
| 7 | PS | Summary of treatment and maintenance plan | Repeat treatment content and establish a maintenance plan to decrease the risk of relapse in exhaustion-related behaviors. | Maintenance plan |
| MD, Medical Doctor; PS, Psychologist; PH, Physiotherapist | | | | |
| **Physical exercise group** | | | | |
| Session | Profession | Content | Purpose | Home Assignment |
| 1 | PH | *Awareness of breathing and bodily exertion* Participants practice stomach breathing and rate their physical effort during varying intensity walks, using the BORG-RPE-scale compared to their pulse measured by a pulse-clock. | Enhance stomach-breathing skills and get a sense between the difference in perceived exertion and actual physiological response (pulse). | Practice stomach breathing and continue using the sig BORG-RPE-scale during physical activity |
|  |  |  |  |  |
| 2 | PH | *Core and weight training* Basic core exercises together with free weights exercises. | Learn a couple of essential core and weight exercises and how to adjust the intensity of these in accordance with current bodily prerequisites | Do the instructed core and weight exercises at least once until the next physical activity session. |
|  |  |  |  |  |
| 3 | PH | *Guide to accessible strength and fitness training*  Walk to an outdoor gym and a run-through of different weight and fitness-exercises available at outdoor gyms. | Learn accessible ways to perform weight and fitness exercises outdoors. Increase awareness on how to continue relaxing muscles not used during physical exertion. | Get information and a map of different outdoor gyms around Stockholm |
| MD, Medical Doctor; PS, Psychologist; PH, Physiotherapist | | | | |
|  | | | | |

*References*

1. Öst LG, Westling BE, Hellström K. Applied relaxation, exposure in vivo and cognitive methods in the treatment of panic disorder with agoraphobia. Behav Res Ther. 1993;31(4):383–94. Available from: http://www.ncbi.nlm.nih.gov/pubmed/8099789
