# Supplementary material for: Standardized multimodal intervention for stress-induced exhaustion disorder: an open trial in a clinical setting
Source: BMC Psychiatry. 2020 Nov 5;20:526. doi: 10.1186/s12888-020-02907-3 (PMC7643309; doi:10.1186/s12888-020-02907-3)
Supplement: Supplementary file 2 — Additional file 2. Extended demography supplement. [file 12888_2020_2907_MOESM2_ESM.docx]

|  |  |  |  |  |  |  |
| --- | --- | --- | --- | --- | --- | --- |
| **Supplementary table with a complete description of baseline demographic variables of patients with Stress-induced Exhaustion disorder (completers and drop-outs; *N* = 390) participating in a 24-week Multimodal intervention** | | | | | | |
| Variable | Total | % | Completed MMR 24 v | % | Drop-outs | % |
| N | 390 | 100 | 379 | 97 | 11 | 3 |
|  |  |  |  |  |  |  |
| Age, mean years | 43,69 |  | 43,66 |  | 44,91 |  |
| - SD | 9,42 |  | 9,46 |  | 8,47 |  |
|  |  |  |  |  |  |  |
| Sex |  |  |  |  |  |  |
| - Female | 344 | 88 | 333 | 88 | 11 | 100 |
| - Male | 46 | 12 | 46 | 12 | 0 | 0 |
|  |  |  |  |  |  |  |
| Marital status |  |  |  |  |  |  |
| - Single | 122 | 31 | 118 | 31 | 4 | 36 |
| - Married/living together | 238 | 61 | 233 | 61 | 5 | 45 |
| - Partner (living apart) | 21 | 5 | 20 | 5 | 1 | 9 |
| - Other | 9 | 2 | 8 | 2 | 1 | 9 |
|  |  |  |  |  |  |  |
| Family situation |  |  |  |  |  |  |
| - Living with children at home | 239 | 61 | 232 | 61 | 7 | 64 |
|  |  |  |  |  |  |  |
| Number of children |  |  |  |  |  |  |
| - 0 | 100 | 26 | 99 | 26 | 1 | 9 |
| - 1 | 61 | 16 | 57 | 15 | 4 | 36 |
| - 2 | 153 | 39 | 150 | 40 | 3 | 27 |
| - 3 | 58 | 15 | 56 | 15 | 2 | 18 |
| - ≥ 4 | 18 | 5 | 17 | 4 | 1 | 9 |
|  |  |  |  |  |  |  |
| Education |  |  |  |  |  |  |
| - Elementary school | 13 | 3 | 11 | 3 | 2 | 18 |
| - Secondary School (2-3 years) | 79 | 20 | 76 | 20 | 3 | 27 |
| - University < 3 years | 62 | 16 | 62 | 16 | 0 | 0 |
| - University ≥ 3 years | 191 | 49 | 185 | 49 | 6 | 55 |
| - Other | 45 | 12 | 21 | 6 | 0 | 0 |
|  |  |  |  |  |  |  |
| Nationality |  |  |  |  |  |  |
| - Sweden | 337 | 86 | 327 | 86 | 10 | 91 |
| - Scandinavia | 11 | 3 | 10 | 3 | 1 | 9 |
| - European | 10 | 3 | 10 | 3 | 0 | 0 |
| - Other | 32 | 8 | 32 | 8 | 0 | 0 |
|  |  |  |  |  |  |  |
| Socioeconomic status* |  |  |  |  |  |  |
| - less than 23 000 €/year | 36 | 10 | 35 | 10 | 1 | 9 |
| - 23 000 € - 46 000 €/year | 132 | 34 | 127 | 34 | 5 | 45 |
| - 46 000 € - 69 000 €/year | 94 | 24 | 91 | 24 | 3 | 27 |
| - 69 000 – 92 000 €/year | 64 | 16 | 63 | 17 | 1 | 9 |
| - more than 92 000 €/year | 62 | 16 | 61 | 16 | 1 | 9 |
|  |  |  |  |  |  |  |
| Occupational status |  |  |  |  |  |  |
| - Employed/self-employed | 365 | 94 | 354 | 93 | 11 | 100 |
| - Studying | 20 | 5 | 19 | 5 | 1 | 9 |
| - Unemployed | 25 | 6 | 25 | 7 | 0 | 0 |
|  |  |  |  |  |  |  |
| Type of work |  |  |  |  |  |  |
| - Handling of heavy material | 7 | 2 | 7 | 2 | 0 | 0 |
| - Heavy repetitive work | 21 | 6 | 19 | 5 | 2 | 18 |
| - Medium repetitive work | 67 | 18 | 64 | 17 | 3 | 27 |
| - Easy repetitive work | 21 | 6 | 21 | 6 | 0 | 0 |
| - Administration/computer work | 249 | 68 | 243 | 64 | 6 | 55 |
|  |  |  |  |  |  |  |
| Approved sick-leave compensation |  |  |  |  |  |  |
| - 0 | 99 | 25 | 95 | 25 | 4 | 36 |
| - 25 | 13 | 3 | 13 | 3 | 0 | 0 |
| - 50 | 60 | 15 | 58 | 15 | 2 | 18 |
| - 75 | 36 | 9 | 36 | 9 | 0 | 0 |
| - 100 | 182 | 47 | 177 | 47 | 5 | 45 |
|  |  |  |  |  |  |  |
| Working time (including studies) | 24,89 |  | 24,68 |  | 31,82 |  |
| - 0 | 232 | 59 | 225 | 58 | 7 | 2 |
| - 1-25 | 34 | 9 | 34 | 9 | 0 | 0 |
| - 26-50 | 71 | 18 | 70 | 18 | 1 | 0 |
| - 51-75 | 16 | 4 | 16 | 4 | 0 | 0 |
| - 76-100 | 37 | 9 | 34 | 9 | 3 | 1 |
|  |  |  |  |  |  |  |
| Symptom duration before seeking treatment |  |  |  |  |  |  |
| - ≤6 months | 76 | 19 | 74 | 20 | 2 | 18 |
| - 7-12 months | 73 | 19 | 70 | 18 | 3 | 27 |
| - > 12 months | 241 | 62 | 235 | 62 | 6 | 55 |
|  |  |  |  |  |  |  |
| Previously on sick-leave due to Stress-induced Exhaustion disorder | 137 | 35 | 133 | 35 | 4 | 36 |
|  |  |  |  |  |  |  |
| Comorbid pain |  |  |  |  |  |  |
| - Suffers from some form of physical pain | 257 | 66 | 249 | 64 | 8 | 73 |
| - Describes longstanding pain | 48 | 12 | 46 | 12 | 2 | 18 |
|  |  |  |  |  |  |  |
| Diagnoses |  |  |  |  |  |  |
| - Number of patients with only SED (43.8A) | 217 | 56 | 209 | 55 | 8 | 73 |
| - Psychiatric comorbidity (F-diagnosis) | 155 | 40 | 152 | 40 | 3 | 27 |
| - Five most common diagnostic codes: F32.9, F32.1 F41.2 F41.9 F33.1 |  |  |  |  |  |  |
| - Somatic comorbidity (M-, R-, N-, T-, G-diagnosis) | 27 | 7 | 27 | 7 | 0 | 0 |
| - Number of patients with more than one comorbid diagnosis | 32 | 8 | 32 | 8 | 0 | 0 |
| - Number of patients with both somatic and psychiatric comorbidity | 15 | 4 | 15 | 4 | 0 | 0 |
|  |  |  |  |  |  |  |
| Medications |  |  |  |  |  |  |
| - Psycholeptic sleep medication (ATC code N05) | 103 | 26 | 101 | 27 | 2 | 18 |
| - Psychoanaleptic antidepressant medication (ATC code N06A) | 158 | 41 | 158 | 42 | 0 | 0 |
| - Psycholeptic sedative medication (ATC code N05) | 72 | 18 | 69 | 18 | 3 | 27 |
| - Other, incl. pain medication, paracetamol/NSAID and medications not prescribed by a physician | 254 | 65 | 252 | 66 | 6 | 55 |
| *The median wage in Sweden is 34 000 €/year, from: Medianlöner i Sverige [Internet]. [cited 2020 Apr 27]. Available from: https://www.scb.se/hitta-statistik/sverige-i-siffror/utbildning-jobb-och-pengar/medianloner-i-sverige/ | | | | | | |
|  |  |  |  |  |  |  |
