# Supplementary material for: Standardized multimodal intervention for stress-induced exhaustion disorder: an open trial in a clinical setting
Source: BMC Psychiatry. 2020 Nov 5;20:526. doi: 10.1186/s12888-020-02907-3 (PMC7643309; doi:10.1186/s12888-020-02907-3)
Supplement: Supplementary file 3 — Additional file 3. Supplementary figures of return-to-work-rates. [file 12888_2020_2907_MOESM3_ESM.docx]

**Supplementary figure 1**

Legend: Distribution of self-reported working time in a sample of patients with Stress-induced Exhaustion disorder participating in a Multimodal intervention, measured at different time points: Pre (*N* = 390), before treatment; Post (*n* = 375), post-treatment; 12MFU (*n* = 341), 12-month follow-up.

**Supplementary figure 2**

Legend: Distribution of self-reported sick-leave compensation in a sample of patients with Stress-induced Exhaustion disorder participating in a Multimodal intervention, measured at different time points: Pre (*N* = 390), before treatment; Post (*n* = 375), post-treatment; 12MFU (*n* = 341), 12-month follow-up.
